# PP78 Data Extrapolation With Survival Curves: An Alternative Approach With Aggregated Data

**DOI:** 10.1017/S0266462324002472

**Published:** 2025-01-07

**Authors:** Ivan Ricardo Zimmermann, Solange Borges

## Abstract

**Introduction:**

Recently, there has been considerable emphasis on survival curves for data extrapolation, especially in the field of economic evaluation in oncology. Common methods for adjusting survival curves are complex and heavily reliant on individual patient data (IPD), which may not be feasible for health technology assessment (HTA). We propose an alternative method for survival curve extrapolation with direct adjustment to aggregated data.

**Methods:**

Common parametric survival analysis models were tested: exponential, Weibull, log-normal, log-logistic, generalized gamma, and Gompertz. We had access to the IPD from a published randomized clinical trial (n=694) testing therapies (anastrozole and fulvestrant) for metastatic breast cancer with 10 years of follow-up on progression-free survival (PFS) and overall survival (OS) outcomes. After adjusting the original IPD, we sought to fit models to published aggregated data (Kaplan–Meier curves) using nonlinear regressions and optimization algorithms. Both methods were compared in terms of visual inspection and statistical fit quality (Akaike information criterion [AIC] and Bayesian information criterion [BIC]).

**Results:**

Survival curves directly adjusted to aggregated data showed a visually similar profile compared to IPD adjustments. According to AIC/BIC values, Weibull and generalized gamma distributions best fit OS data, both in individualized and aggregated approaches. For PFS, log-logistic and log-normal curves were the best choices for the anastrozole arm, and for fulvestrant, the best choices were log-normal and generalized gamma for individualized data, and Gompertz and generalized gamma for the aggregated method. The proposed R language code proved to be reproducible and amenable to automation in future HTA applications.

**Conclusions:**

Directly adjusting survival curves to aggregated data is a simple and useful alternative in situations where access to IPD is not feasible.

